# A One-Stage Approach for Surface Anomaly Detection with Background Suppression Strategies

**DOI:** 10.3390/s20071829

**Published:** 2020-03-25

**Authors:** Gaokai Liu, Ning Yang, Lei Guo, Shiping Guo, Zhi Chen

**Affiliations:** School of Automation, Northwestern Polytechnical University, Xi’an 710129, China; lgk@mail.nwpu.edu.cn (G.L.); lguo@nwpu.edu.cn (L.G.); spguo@nwpu.edu.cn (S.G.); cz@mail.nwpu.edu.cn (Z.C.)

**Keywords:** surface anomaly detection, computer vision, deep learning, one stage, background suppression

## Abstract

We explore a one-stage method for surface anomaly detection in industrial scenarios. On one side, encoder-decoder segmentation network is constructed to capture small targets as much as possible, and then dual background suppression mechanisms are designed to reduce noise patterns in coarse and fine manners. On the other hand, a classification module without learning parameters is built to reduce information loss in small targets due to the inexistence of successive down-sampling processes. Experimental results demonstrate that our one-stage detector achieves state-of-the-art performance in terms of precision, recall and f-score.

## 1. Introduction

Surface defect inspection plays a vital role in manufacturing processes for product quality control. It is used throughout many different domains, such as metal [1,2,3], concrete [4], plastic [5], or fabric [6,7] surface anomaly detection. However, these tasks are often carried out manually, requiring specialized skills to be mastered, which is quite inefficient and limits the rapid development of relevant industrial applications. Therefore, automatic defect detection is highly necessary and has more importance than ever for quality control in industrial processes.

With the development of computer vision technologies, numerous studies have been devoted to the methods for detect surface defects. Generally, these approaches can be divided into two main categories: (i) classical methods based on DCT models [8], histograms of oriented gradients (HOGs) [9], etc. When it involves manual features, on the one hand, these features are difficult to select or design for adoption in a specific application, and on the other hand, they are not universal for multiple different tasks and (ii) Deep learning model-based methods. More and more excellent convolutional neural network models (CNN) have been emerging in the past few years, such as ResNet [10], PSPNet [11], YOLO [12], FCN [13], and DeepLabv3+ [14]. Moreover, FCN combines deep features with more shallow ones by transposed convolution operations to acquire both semantic information and more low-level information. Attention U-Net [15] is proposed to enhance foregrounds by using a multiplication mechanism in adjacent stages between encoder and decoder. The main contributions of Network in Network [16] is that the global average pooling layer replaces the fully connected layer, the average value of the output feature maps reflects the category of the image, and then the probability of each category is obtained by a softmax layer.

Early work where convolutional neural networks are applied in surface anomaly inspection is described in [17], which proposed a convolutional neural network based on maximum pooling for steel defect classification. It showed that no hand-engineered features were used but better results were obtained. However, since no non-linear activation functions are utilized, the network cannot carry out deep feature extraction. Therefore, the performance of the network is greatly limited. With the emergence of the activation function RELU, most subsequent networks used it to deepen the network and obtain rich non-linear features. Natarajan et al. [18] proposed a multi-layer depth feature extraction as part of the classification framework of small data sets via transfer learning. The flexibility of this method can enable any convolutional neural network to be used for feature extraction. Moreover, a linear support vector machine classifier based on majority voting mechanism (MVM) was proposed to overcome the overfitting problem of small data sets by fusing multi-layer depth features. García et al. [19] presented a method that explores optimal configurations to obtain the possible best network which includes the preparation of the dataset, selection of the initial methods, and exploration of the hyper-parameters to acquire the appropriate configuration. This method is applicable to tasks involved in CNN-based defect detection system. Aiming at various forms and types of defects appear in the surface of steel during metal forming process, Youkachen et al. [20] put forward an unsupervised learning model based on convolutional AutoEncoder. The model employs Cascade AutoEncoder (CAE) to reconstruct the defects, and then these defects are segmented through a sharpening process, which is based on the assumption that the reconstructed images contain only normal features. Jing et al. [21] presented a fabric anomaly inspection algorithm, which can detect various kinds of fabric defects. It’s worth mentioning that it not only directly utilizes the original images as input, but it also divides the fabric images into multiple patches along the natural cycle of the fabric surface as the operation objects to train a CNN model. 

These works above can represent two different one-stage methods for surface anomaly detection which can be divided into classification and transfer learning methods. However, classification methods are not appropriate for the small datasets studied in this paper as overfitting is prone to occur, and transfer learning is not suitable due to the large gap between the images of pre-training network and the KolektorSDD dataset used in this paper, which has been already proved experimentally in the comparative literature [5]. In addition, the method where only a segmentation network is used can be considered to be applicable for small datasets as it can largely alleviate overfitting problems, but such methods are not currently popular for surface anomaly detection and classification, and we hold that the complex background interference is a significant factor for it.

In order to improve the accuracy of anomaly surface classification in small sample sets, two-stage approaches, that is, ones where both the segmentation network and the classification network need to be trained, have been popular in recent years. Racki et al. [22] proposed a compact CNN-based framework which included segmentation and decision networks for surface anomalies. The segmentation network was firstly trained, and then all the parameters were frozen, which was followed by the training of the classification network to a high classification accuracy. The approach introduced by Tabernik et al. [5] is an extended version of the model [22], and the main difference lies in that several convolution and pooling operations are added to the classification network part. Since a larger perceptive field is acquired, the semantic information is further enhanced. Song et al. [23] designed a two-stage network which reduces the complexity of the network and where global information is more easily captured owing to the utilization of residual and squeeze-and-excitation network. Specifically, a residual module decreases the depth of segmentation networks, a squeeze section aggregates spatially distributed information, and an excitation operation acquires a channel-wise dependency relationship using non-linear functions.

For the two-stage methods above, the segmentation network can alleviate overfitting issues and then the classification module makes use of the fine features from the segmentation network to further improve the accuracy of classification. However, small objects cannot be well represented by the features before a fully connected layer due to continuous pooling and convolution operations, as well as the lack of decoders.

Compared to the aforementioned works, the proposed one-stage method is applied not only to suppress background patterns, but also capture well the underlying defects, and it is verified that our approach can achieve better performance in precision, recall and f-score in KolektorSDD dataset than the latest method [5]. The main contributions of this paper are as follows:

(1) From the overall framework, the features of small targets can be well represented. Compared with the state-of-the-art methods, this paper not only introduces a decoder, but the classification module with continuous down samplings and convolutions are replaced only by calculating the pixel average values of segmentation outputs, which decreases the information loss of small targets.
(2) Coarse and fine background suppression modules are designed at the decoder of the segmentation network, which expands the feature difference between positive and negative samples. To the best of our knowledge, a module designed with iterative multiplication in fine background suppression method has not been available before.
(3) Due to the absence of a complex optimization process and learning parameters for the classification module in this paper, the work load is lightened for industrial tasks.

The remainder is specifically organized as follows: The overall methodology is described in Section 2, and in Section 3, a quantitative comparison is experimentally demonstrated and different aspects of the results of the two models are discussed. Finally, we conclude this paper in Section 4.

## 2. Materials and Methods

### 2.1. Overview

**Figure 1:**
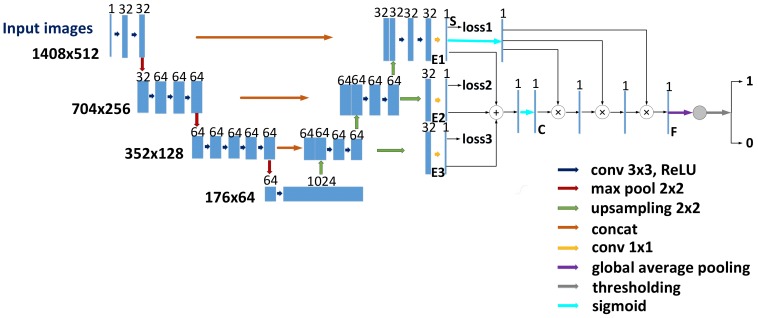
Proposed one-stage model for surface defect detection.

The underlying hypothesis behind our architecture relies mainly on two aspects: (i) background suppression is conducive to boosting the classification process. (ii) due to successive down-sampling processes and the lack of decoders, it is difficult for two-stage methods to fully describe the features of small anomalies. In addition, we argue that f-score, precision and recall are more appropriate quantitative metrics than average precision for surface crack detection in industry, and the corresponding reason is explained in Section 3. Our experiments demonstrate the suggested method is effective and outperforms that proposed in the latest paper. Figure 1 shows the overall structure of the method. 

### 2.2. Segmentation Module

For segmentation tasks, the network is trained with a pixel-wise loss, which can effectively consider each pixel as an individual training sample to give each pixel a meaning of specific classification. This means that the effective number of training samples is largely increased for small sample set, therefore the overfitting issues can be greatly alleviated [5].

#### 2.2.1. Encode-Decoder Network

**Figure 2:**
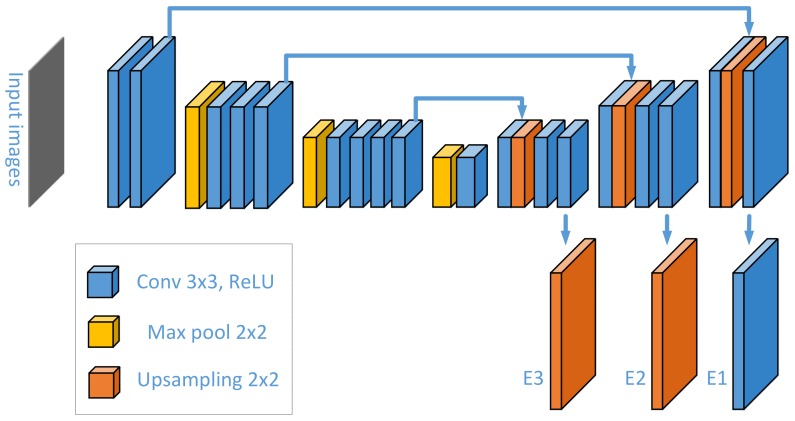
Encode-decoder network.

In the segmentation module, each pixel in the images is soft-classified in the form of a probability. The segmentation network described in [5] is used as encoder in this paper, and decoders are added and follow the last convolution layer of the encoder, meanwhile, skip connections are also utilized in a similar manner as in U-Net [24]. There are three upsampling operations which split the decoder process into three consecutive stages. One transposed convolution layer is followed by two 5 × 5 convolution layers in each stage. The decoder and encoder are merged by a concatenation operation as seen in Figure 2. Since defect and background patterns have different feature representations, the encode-decoder structure above serves as an underlying feature extractor to find the specific patterns of surface defects. E1, E2 and E3 represent the feature maps after up-sampling for the last ones of three stages from bottom to second top.

#### 2.2.2. Coarse Background Suppression

**Figure 3:**
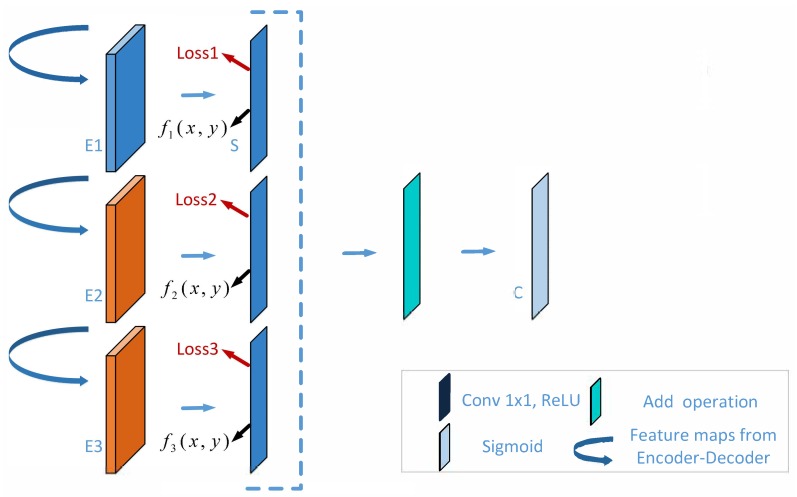
Coarse background suppression (CBS).

A deep supervision mechanism [25] is introduced to our coarse background suppression module is shown in Figure 3. The last convolution output of each stage in the decoder block is up-sampled to the same size as input images, and then, these feature maps are subjected to 1 × 1 convolution for dimension reduction. We selected the first three of the highly fused features from bottom to second top. On the one hand, they are subjected to a sigmoid function and intermediate supervision sequentially to acquire more semantic outputs than the bottom layer. On the other hand, these single-channel features are merged via an add operation followed by a sigmoid function to suppress most background noises, which can be formulated as:

(1) $$C\left(i,j\right)={\left(1+e^{-\sum_{k=1}^{3}f_{k}(i,j)}\right)}^{-1}.$$

Here $f_{k}(x,y)$ denote the value of the single-channel feature map after E1, E2 and E3 are implemented by 1 × 1 convolution. C(i,j) signifies the outputs of coarse background suppression, and three stages from bottom to second top are selected via experiments.

Loss1, Loss2 and Loss3 refer to objective functions concerning $F_{1}(x,y)$, $F_{2}(x,y)$ and $F_{3}(x,y)$ with ground truth, respectively. Here, $F_{1}(x,y)$, $F_{2}(x,y)$ and $F_{3}(x,y)$ are calculated by sigmoid function for $f_{1}(x,y)$, $f_{2}(x,y)$ and $f_{3}(x,y)$, that is:

(2) $$F_{k}(x,y)=\frac{1}{1+e^{-f_{k}(x,y)}}$$

Therefore, Loss1, Loss2 and Loss3 in Figure 3 can be expressed in terms of $\xi_{1}$, $\xi_{2}$ and $\xi_{3}$ as follows:

(3) $$\xi_{k}=-\frac{1}{n}\sum_{i=1}^{n}\left\{H(x,y)logF_{k}(x,y)+[1-H(x,y)]log[1-F_{k}(x,y)]\right\}$$

**Figure 4:**
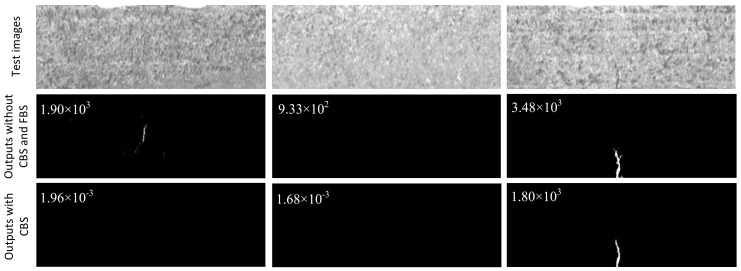
Examples with coarse background suppression or not. Negative, negative, positive samples are shown, respectively, in row 1, while the two images on the left in row 2 signify two kinds of prediction results of negative samples with an obvious false alarm or not, and the figures on the top left denote the scores via our classification module.

Here *H*(*x*,*y*) signifies the pixel value of the ground truth. As semantic information gets stronger, background noises can be more correctly distinguished, so the corresponding feature values in high layers are quite small. In this case, therefore, an add operation of the bottom and high feature maps makes most of background noises that appear in the bottom layer tend to be pulled back to the low saturated area by high-level information. From the examples shown in Figure 4 we can see that the obvious noise blocks disappear after the coarse background suppression strategy is employed, and in addition, apart from the obvious false alarm information, other background patterns are also largely suppressed, while there is just a small reduction for defects.

#### 2.2.3. Fine Background Suppression

**Figure 5:**
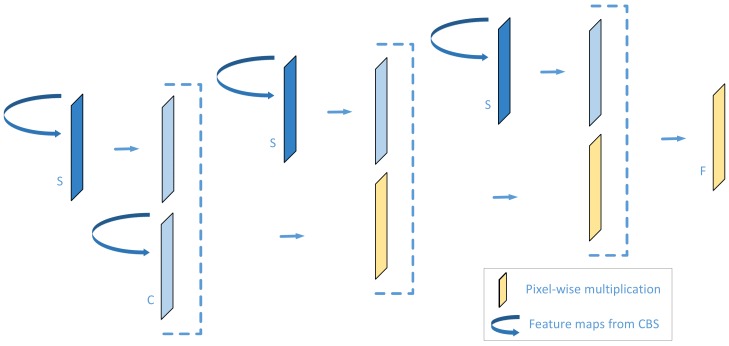
Fine background suppression (FBS).

**Figure 6:**
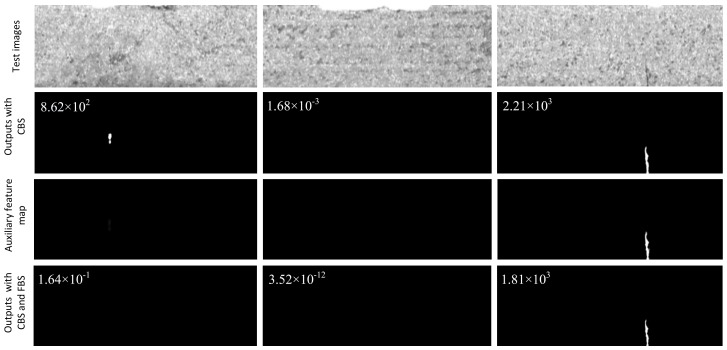
Examples with fine background suppression or not. Negative, negative, positive samples are shown respectively in row 1, where the first image in row 1 represents a few new prediction results with obvious false alarms via coarse background suppression, while the images in row 3 indicate auxiliary feature maps from bottom layers, and the figures in the top left corners denote the scores via our classification module.

There are still some noise patterns that cannot be weighed down or removed by the strategy described above. Inspired by the model proposed in [15], we further design the fine background suppression module shown in Figure 5. After the coarse stage above, erroneous judgments that there exist strong defect patterns in corresponding positions could be made by the fusion feature with sigmoid function. However, we notice that there are several defect-free or weak false defect patterns (Figure 6) which appear in the predicted mask of the bottom layer.

Therefore, in our fine background suppression module, the merged result from the coarse background suppression section is integrated with the bottom output in an iterative manner via pixel-wise multiplication performed three times to further cut down false alarm rate, which can be expressed as:

(4) $$F\left(i,j\right)=C\left(i,j\right){\left(1+e^{-f_{1}(i,j)}\right)}^{-3}.$$

Here $f_{1}(x,y)$ indicates the values of the single-channel feature map after E1 is executed by 1 × 1 convolution. F(i,j) represents the output of fine background suppression.

As the iteration progresses, the two background patterns presented in the case like Figure 6 decrease significantly while the predicted surface defects result is almost constant or displays a limited change. Therefore, surface defects and background patterns can be further distinguished with the refine mechanism.

### 2.3. Classification Module

**Figure 7:**
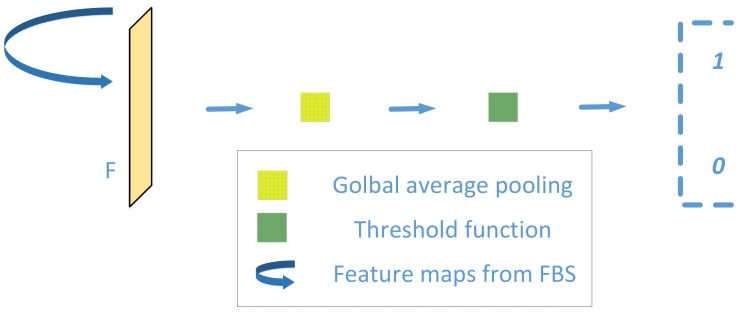
Classification Module.

The classification module consisting of a global average pooling and threshold function is shown in Figure 7, which can classify the experimental images into specific categories based on the segmentation results above. The mean value of every channel can be calculated with global average pooling, which is put forward in [16]. That is to say, each channel is given a category feature while spatial information is aggregated. Moreover, as for pooling itself, there are no parameters to learn in this process. Therefore, it is quite dependent on the segmentation result if global average pooling is used as classifier. However, as a result of the coarse and fine background suppression mechanism above, the proposed segmentation method is easily compatible with global averaging pooling which is utilized as a direct classifier. Furthermore, as the output of global average pooling presents in the manner of mean probability, a threshold module is added followed by global average pooling to meet the needs of industrial applications, which can be described as:

(5) $$G=\sum_{i=1}^{M}\sum_{j=1}^{N}I\left(i,j\right).$$

(6) $$L=\left\{\begin{array}{c} 1,\;\;G\leq \sigma \\ 0,\;\;G>\sigma \end{array}\right..$$

Here *I*(*i*,*j*) represents the pixel value of segmentation outputs, and *M*, *N* denote the size of the images. σ signifies a preset threshold value. G and *L* signify the pixel-value sum of the segmentation outputs and classification results, respectively.

**Figure 8:**
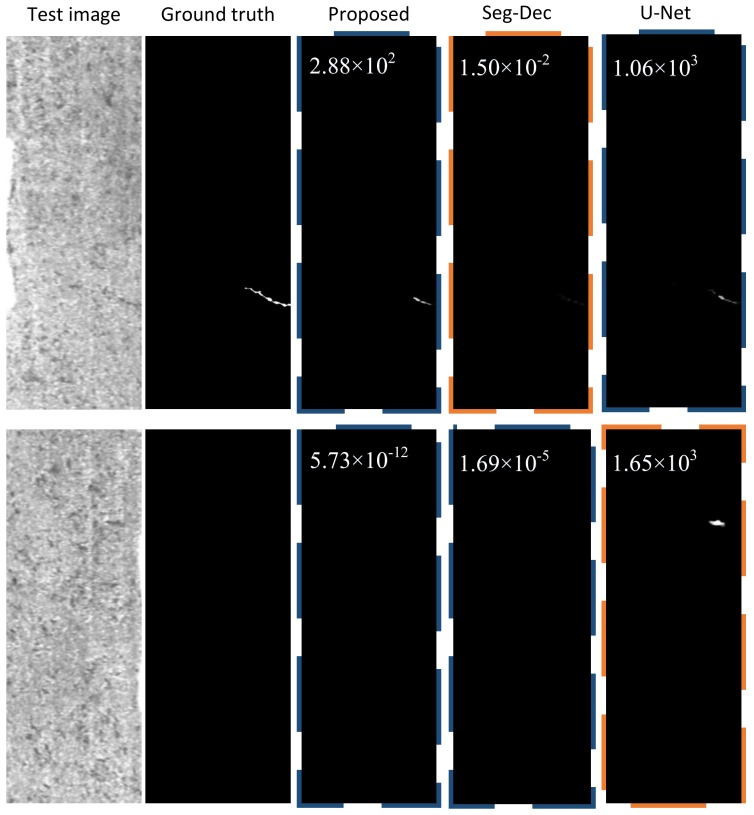
Examples of comparison experiments. Blue dashed border denotes true positive (TP) and true negative (TN), and orange dashed border signifies false positive (FP) and false negative (FN).

Considering that the classification module has no training process, the proposed surface anomaly detection approach can be referred as a one-stage method, where we just train the segmentation section. Figure 8 illustrates two classification examples using the comparative approaches described in this paper.

## 3. Results and Discussion

We evaluate the performance of our method on the KolektorSDD dataset [5], which contains 399 surface microscopic images of plastic embedding materials in electrical commutators. There are 52 images with fractions or cracks while 347 images are without any defects. For the sake of comparison, the dataset is divided into three subsets the same as in the comparative method [5] for 3-fold cross validation, all the images are also uniformly scaled to 1408 × 512, and the experiment is carried out with five different labels as well.

From the perspective of quantitative analysis, in this paper, we adopt f-score, precision and recall instead of the average precision used in [5], out of the consideration that average precision (AP) is defined as:

(7) $$AP=\sum_{n}\left(R_{n}-R_{n-1}\right)P_{n}.$$

where *R_n_* and *P_n_* are the recall and precision at the *n*-th threshold. From Equation (7) we can see that as a comprehensive index under different thresholds, average precision does not accord with the reality of industrial production. Actually, the maximum f-score with a certain fixed threshold in the whole dataset is more appropriate than average precision, as it is not important for the non-maximal f-score under other thresholds in industrial scenarios, which is quite similar with the sense of the ODS criterion in segmentation tasks [26].

The precision, recall and f-score (*F*) values we reported in the following experiment can be calculated by true positive (*TP*), false positive (*FP*), false negative (*FN*), and *β*, i.e.:

(8) $$Precision=\frac{TP}{TP+FP}.$$

(9) $$Recall=\frac{TP}{TP+FN}.$$

(10) $$F_{\beta }=(1+\beta^{2})\frac{Precision\times Recall}{\beta^{2}(Precision+Recall)}.$$

where *F_β_* is a weighted harmonic average value [27] of precision and recall with non-negative weight *β*, and *β*^2^ = 1 is set to treat recall with equal importance as precision in this paper.

**Figure 9:**
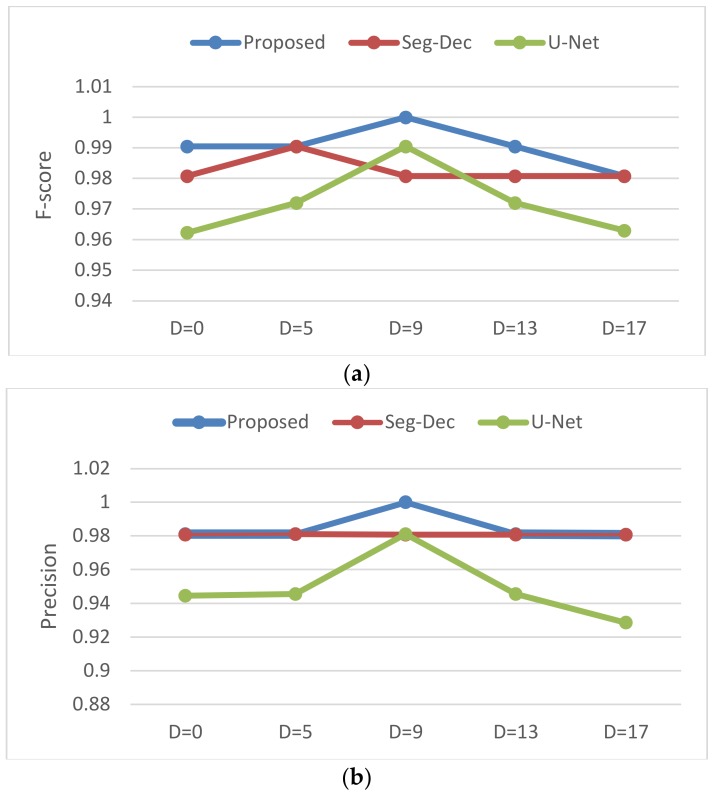

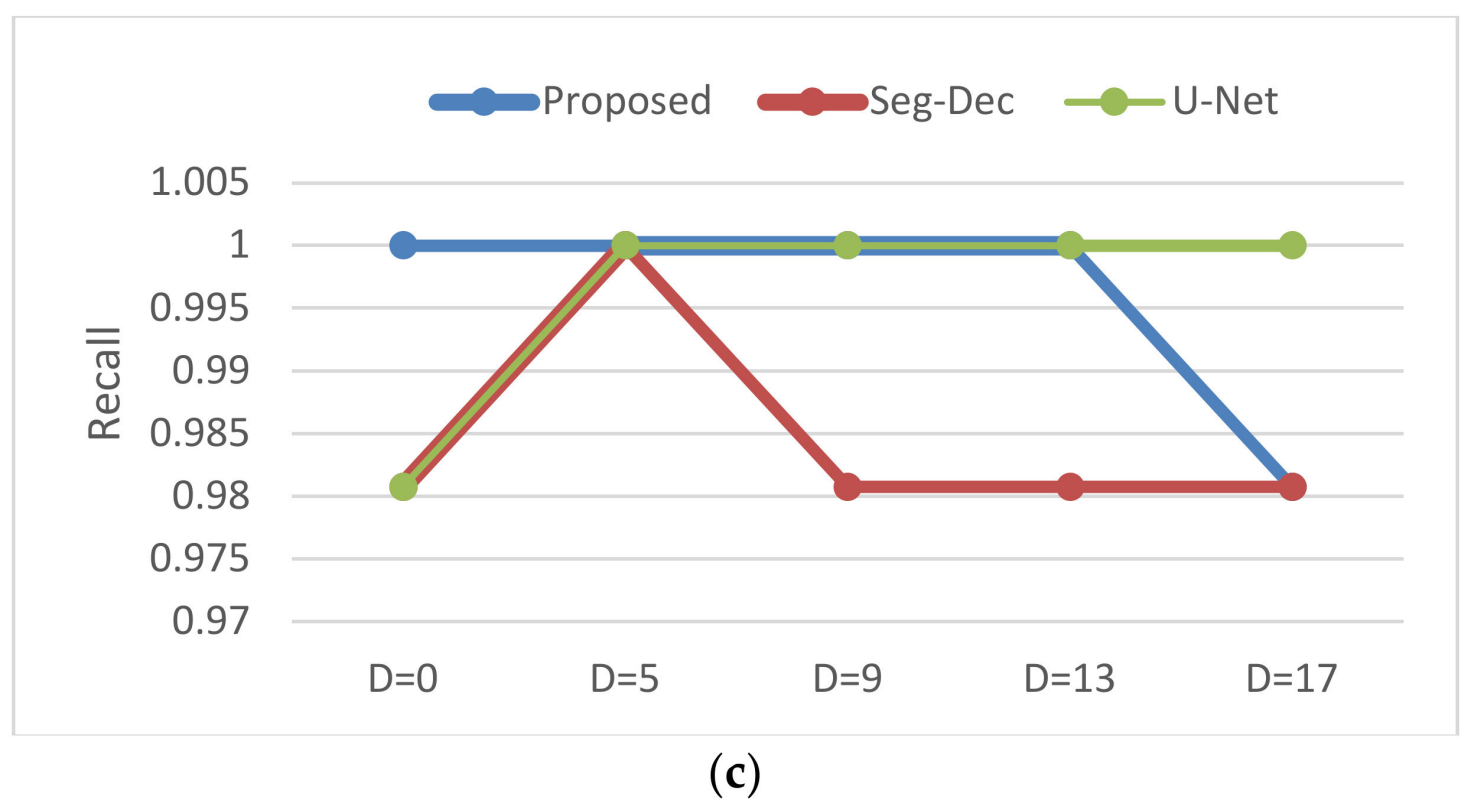
Performance comparisons with different dilate rate. (**a**) f-score; (**b**) precision; (**c**) recall.

We compare the suggested method with the state-of-the-art approach called the Seg-Dec network and the classical segmentation network U-Net on the KolektorSDD dataset. In the Seg-Dec network, all the parameters are frozen after the segmentation network is trained, then the decision layers are trained independently based on this frozen segmentation network. In the U-Net network, transposed convolutions are employed in the upsampling process, and the segmentation output is followed by our classification module to achieve final category prediction. Through experiments, the threshold values are set as 1000, 1350, 1300, 1250, 1250 owing to the different mean value of background patterns under different labels with dilation rate 0, 5, 9, 13, 17. While in our approach, coarse and fine background suppression strategies are introduced into the segmentation framework and only the segmentation network is trained, and the output is fed into the global average pooling operation in classification module, which is followed by a threshold function to achieve final classification. The segmentation network is trained via an Adam optimizer with cross-entropy loss, a learning rate of 0.001 is used, the batchsize is set to 1, and the threshold value is set as 100 to decide whether there exist defects or not. Figure 9 shows the detailed classification results of the three methods under labels with five different dilation (D) rate.

**Figure 10:**
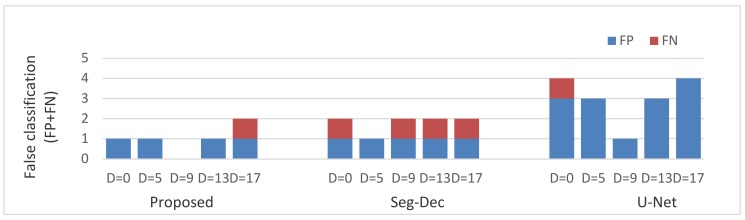
FP and FN comparisons with different dilate rate.

From Equations (8) and (9) we can see that FP and FN reflect precision and recall from a more intuitive perspective, therefore, apart from f-score, precision and recall, FP and FN, which are shown in Figure 10, are also reported for our quantitative analysis. 

As revealed in Figure 9 and Figure 10, compared to the Seg-Dec network, our approach yields a larger f-score on three labels and equivalent results on the others, and a similar phenomenon occurs in metric precision and recall. It is a remarkable fact that 100% of f-score, precision and recall can be realized via the proposed model when the dilation rate is 9. Moreover, we achieve that no FN happens in four circumstances while only one in the Seg-Dec method, which means that our method is more in line with the needs of industrial applications, after all, false alarms are better than missing detections in most industrial tasks. Compared to the U-Net network, our method can also achieve few quantity of FN, but less FP tend to occur simultaneously. Therefore, it helps to save the working time of removing false alarms.

For the Seg-Dec network, we hold that small defects cannot be well described by the features before the fully connected layer due to continuous pooling and convolution operations in the classification network, and the lack of decoders is another reason, while in our method, for one thing, we carry out the classification process by a global average pooling operation and threshold function instead of using a classification network with continuous down samplings and convolutions, which reduces information loss of small targets, as illustrated in Figure 8, and for another thing, the dual background suppression strategies play a significant part for classification result, as seen in Figure 4 and Figure 6. It is also worth mentioning that the authors also attempted to introduce auxiliary loss to the segmentation module, however, worse results have been obtained. This is explained by the fact that due to the different scope of segmentation and classification, it is difficult for the weight distribution of two loss functions to achieve maximum joint optimization. Nevertheless, the problem is avoided as a result of the classification module without training process in the proposed method. For the U-Net network, few missing detections can be achieved the same as ours owing to the decoders, but it is more prone to yielding false alarms in this case, as illustrated in Figure 8, while our method can improve the problem of false alarms due to the dual background suppression strategies, and meanwhile few missing detections occur. In addition, due to the large feature difference between the anomalies and background patterns under dual background suppression mechanisms, it is beneficial to the threshold setting and the distinction between positive and negative samples.

**Figure 11:**
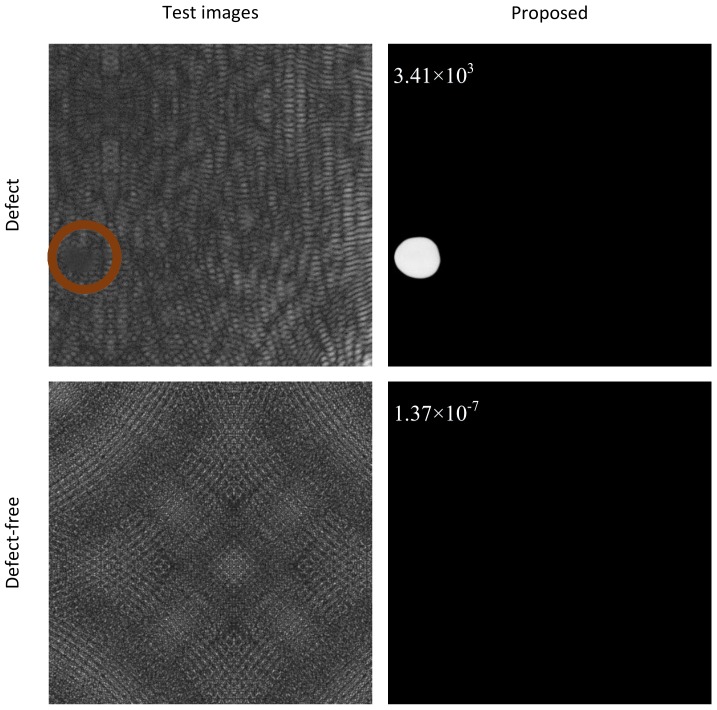
Examples on the DAGM 2007 dataset. The defect is marked in deep orange.

From the experiments on the KolektorSDD dataset described above, we can see that the complex features of targets and even small anomalies can be well described, and the background patterns can be well suppressed as well through our methods. The proposed method can be also extended to defect-detection tasks on the DAGM 2007 dataset (the DAGM 2007 dataset is publicly available at https://hci.iwr.uni-heidelberg.de/node/3616). It consists of 10 datasets, which represent miscellaneous defects on various background textures. We use 108 images as training samples in class 1 and Figure 11 shows two examples of defect detection. The experimental results demonstrate that our method also can enlarge the feature difference between the defects and background patterns and has a strong detection capability for defects on textured backgrounds.

## 4. Conclusions

This paper put forward a one-stage method to improve surface defect detection results from the perspective of specific industrial production. The defect inspection task is converted into segmentation and classification problems. Dual background suppression strategies expand the feature differences between positive and negative samples, and our classification module with no learning parameters decreases the information loss of small defects. Our experiments verified that the suggested model can achieve better results than the latest approach on the KolektorSDD dataset. Furthermore, the proposed method can be expected to be further applied in other similar applications.
